# Late-Onset Glycogen Storage Disease Type II (Pompe's Disease) with a Novel Mutation: A Malaysian Experience

**DOI:** 10.1155/2014/926510

**Published:** 2014-06-30

**Authors:** Hiew Fu Liong, Siti Aishah Abdul Wahab, Yusnita Yakob, Ngu Lock Hock, Wong Kum Thong, Shanthi Viswanathan

**Affiliations:** ^1^Department of Neurology, Kuala Lumpur General Hospital, Jalan Pahang, 50586 Kuala Lumpur, Malaysia; ^2^Molecular Diagnostics and Protein Unit, Institute for Medical Research (IMR), Jalan Pahang, 50586 Kuala Lumpur, Malaysia; ^3^Department of Genetics, Kuala Lumpur General Hospital, Jalan Pahang, 50586 Kuala Lumpur, Malaysia; ^4^Department of Pathology, University of Malaya, Lembah Pantai, 50603 Kuala Lumpur, Malaysia

## Abstract

Pompe's disease (acid maltase deficiency, glycogen storage disease type II) is an autosomal recessive disorder caused by a deficiency of lysosomal acid *α*-1,4-glucosidase, resulting in excessive accumulation of glycogen in the lysosomes and cytoplasm of all tissues, most notably in skeletal muscles. We present a case of adult-onset Pompe's disease with progressive proximal muscles weakness over 5 years and respiratory failure on admission, requiring prolonged mechanical ventilation. Electromyography showed evidence of myopathic process with small amplitudes, polyphasic motor unit action potentials, and presence of pseudomyotonic discharges. Muscle biopsy showed glycogen-containing vacuoles in the muscle fibers consistent with glycogen storage disease. Genetic analysis revealed two compound heterozygous mutations at c.444C>G (p.Tyr148∗) in exon 2 and c.2238G>C (p.Trp746Cys) in exon 16, with the former being a novel mutation. This mutation has not been reported before, to our knowledge. The patient was treated with high protein diet during the admission and subsequently showed good clinical response to enzyme replacement therapy with survival now to the eighth year.* Conclusion*. In patients with late-onset adult Pompe's disease, careful evaluation and early identification of the disease and its treatment with high protein diet and enzyme replacement therapy improve muscle function and have beneficial impact on long term survival.

## 1. Introduction

Glycogen storage disease type II (Pompe's disease) is an autosomal recessive lysosomal storage disease caused by a deficiency of acid *α*-1,4-glucosidase (GAA; acid maltase, EC; 3.2.1.20/3), which is a key enzyme in hydrolyzation of lysosomal glycogen to glucose. Affected individuals will have excessive accumulation of glycogen in the lysosomes and cytoplasm of all tissues, most notably in skeletal muscles. Pompe's disease has a wide spectrum of clinical presentations. The 3 major clinical forms are infantile, juvenile, and adult onset. Infantile form is characterized by rapidly progressive proximal myopathy and cardiomyopathy. Respiratory and cardiac failures are the main causes of death within the first 2 years of life. In contrast, adult-onset Pompe's disease is a slowly progressive disease of proximal myopathy with raised creatine kinase and later involvement of the respiratory muscles, resulting in respiratory failure. Cardiac involvement and it's complications are rare [1, 2].

The deficiency of *α*-1,4-glucosidase results from the mutations in the gene encoding the acid *α*-1,4-glucosidase (GAA gene). GAA gene is located on chromosome 17q25.2-q25.3, approximately 20 kbp long, and consists of 20 exons [3]. To date, more than 300 mutations in the GAA gene have been described in the Pompe's Disease Mutation Database and Human Gene Mutation Database (HGMD). However, data on the genetic mutation of adult-onset Pompe's disease from South East Asia specifically Malaysian patients of Chinese origin is limited. In this paper, we present a case of a novel mutation of adult-onset Pompe's disease in a Malaysian Chinese patient.

## 2. Case Report

A 28-year-old Chinese lady was admitted to the Neurology Service of Kuala Lumpur Hospital in December 2005 with progressive proximal muscle weakness over 5-year duration. Two weeks prior to admission, she had worsening bilateral upper and lower limbs weakness with reduced effort tolerance and breathing difficulties, rendering her unable to walk. Within a day, her breathing worsened necessitating assisted ventilation. At this point, she was treated for a postinfectious polyneuropathy. Family history did not reveal any consanguinity and birth history was unremarkable. She is the youngest of three siblings.

**Figure 1:**
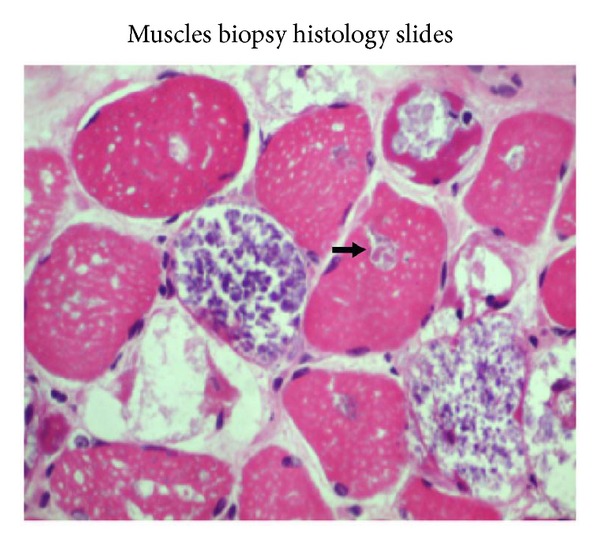
Hematoxylin and Eosin (H&E) staining shows vacuolar myopathy.

**Figure 2:**
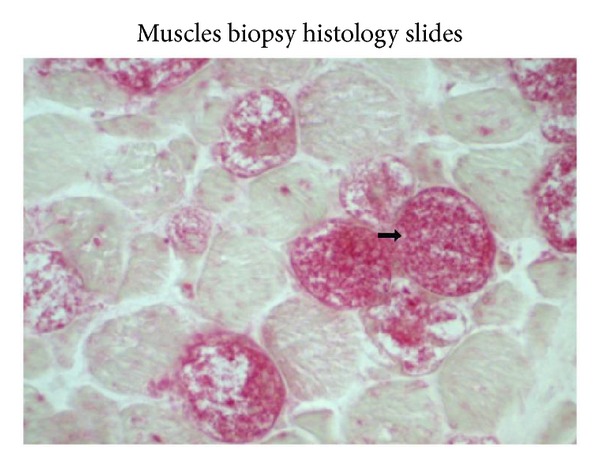
Vacuoles are stained with periodic acid Schiff (PAS).

On admission, neurological examination revealed weak neck flexor muscles with Medical Research Council (MRC) score of 0-1/5. There was marked involvement of the proximal muscles of the lower extremities with MRC score of 1-2/5 compared to the upper extremity shoulder abductors, which had an MRC score of 4/5 bilaterally. Reflexes were intact and plantars were downgoing bilaterally. Chest expansion was poor and the patient required prolonged ventilatory support from the time of admission. Laboratory studies revealed elevated creatine kinase (CK) of 671 U/L and alanine transaminase (ALT) levels of 77 U/L. Electrocardiogram and echocardiogram were normal. Sensory and cranial nerve examination was within normal limits. Nerve conduction and electromyography studies showed evidence of myopathic process with small amplitudes, polyphasic narrow motor unit action potentials (MUAP's), and presence of pseudomyotonic discharges. Muscle biopsy on Hematoxylin and Eosin stain showed glycogen-containing vacuoles in the muscle fibers consistent with glycogen storage disease. The vacuoles were strongly positive for the periodic acid Schiff (PAS) stain (refer to Figures 1 and 2). Acid alpha-glucosidase enzyme activity testing using Guthrie Card alpha-glucosidase assay on a blood spot showed low levels of alpha-glucosidase of 0.04 umol/h/L (reference: 0.3–3.0 umol/h/L).

**Figure 3:**
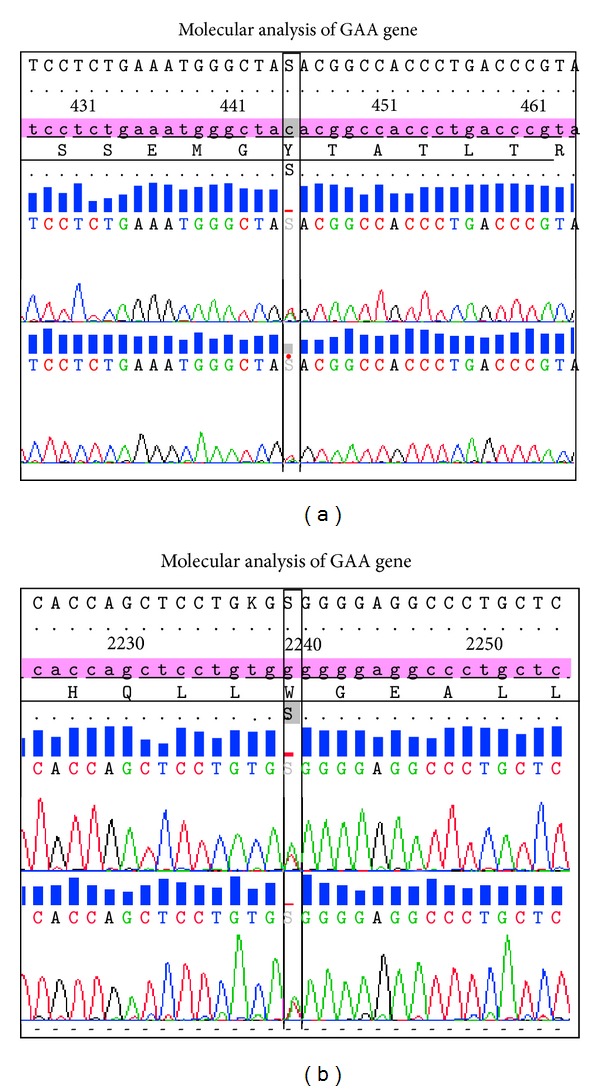
DNA sequence electropherograms of the two heterozygous compounds detected in this patient. The c.444C>G (p.Tyr148*) mutation is identified in exon 2 and c.2238G>C (p.Trp746Cys) mutation is identified in exon 16 of the GAA gene.

**Figure 4:**
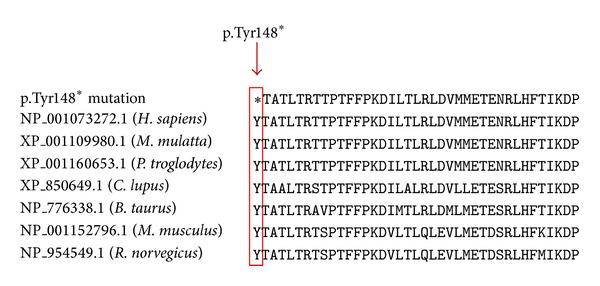
Multiple sequence alignments of GAA homologous sequences in different species are shown. Nonsense mutation reported in this study is highlighted by a red rectangle, illustrating that the p.Tyr148* mutation is in a highly conserved region.

Molecular analysis of the DNA revealed two compound heterozygous mutations at c.444C>G (p.Tyr148*) in exon 2 and c.2238G>C (p.Trp746Cys) in exon 16. The first mutation at c.444C>G showed a nucleotide change at codon 148 introducing a premature stop codon. This mutation disrupts the normal splicing and is predicted to produce a truncated acid *α*-glucosidase. This mutation described is a novel mutation and has not previously been reported in both HGMD and Pompe's Disease Mutation Databases. Homology analysis of the novel mutation showed that the location of the mutation at codon 148 is in a highly conserved region (Figure 3). The second mutation at c.2238G>C causes a change from nonpolar aromatic Tryptophan to polar aliphatic Cysteine at codon 746 and has been known to affect the enzymatic function of acid *α*-glucosidase [4] (refer to Figure 4).

She was started in high protein dietary regime (1.2–1.5 gm of protein/kg/day) with continuous physiotherapy and rehabilitation support. Due to respiratory muscle weakness and diaphragmatic paresis, she was ventilated for a total duration of 9 months. This was complicated with recurrent lung collapses. After 6 months of a high protein dietary regime, she was successfully weaned off from the ventilator with a permanent tracheostomy, supplemented by home oxygen using a concentrator. Upper limb muscle power improved to 5/5 on MRC scale. Neck and proximal lower limbs muscle power improved to 3-4/5 on MRC scale. On discharge, she was able to ambulate unaided for short distances of 140 meters in 6 minutes and independent of activities of daily living. Her creatine kinase level remained elevated at 691 U/L.

The patient was subsequently started on intravenous infusions of recombinant human acid *α*-glucosidase (rhGAA; 20 mg/kg body weight) every 2 weeks. After initial 8 courses of rhGAA therapy over 4 months in 2007, her muscle strength remained stable as on discharge but there was improvement in timed 6-minute walk from 140 meters to 168 meters. Serum creatine kinase level was significantly reduced from 746 U/L before enzyme replacement to 92 U/L. Alanine transaminases also showed marked improvement from 72 U/L to 28 U/L after enzyme replacement therapy (ERT). Despite improvement in creatine kinase level, liver enzymes, and ambulatory status in her 6-minute walking test, her motor performance remained clinically the same. However, her respiratory status continued to decline, requiring noninvasive ventilation in the year 2008. In 2009, enzyme replacement therapy was briefly interrupted due to a worldwide shortage. In 2010, proximal upper limb muscle power remained at 4-5/5 on MRC scale. However, proximal lower limbs muscle power had reduced to 3/5 on MRC scale after the period of discontinuation highlighting the benefit while the patient was on the regular infusions. Distal upper and lower limb power remained full. Her 6-minute walking test was 20 meters due to the needs of noninvasive ventilation occurring after the period of discontinuation of enzyme replacement therapy of one year. Her ability to ambulate reduced over this period necessitating walking aids or assistance and supplementation with a wheelchair. At the present time, she continues to survive on minimal requirement noninvasive ventilation with stable respiratory status as long as her enzyme replacement therapy is not interrupted. Motor performance and ambulatory status have remained stable on 2-weekly enzyme replacement therapy over the last 4 years.

## 3. Discussion

Pompe's disease is an inherited autosomal recessive disorder caused by a deficiency of the lysosomal enzyme acid alpha-glucosidase (GAA). The phenotypic expression occurs when both alleles of the GAA gene harbor a pathogenic mutation and in this case as shown on genetic analysis as described earlier. Data on genetic mutations for late-onset Pompe's disease (childhood, juvenile, and adult-onset) among Chinese populations are limited, with only a handful of reports from China [5] and Taiwan [4]. Most studies analysed patients with infantile-onset Pompe's disease. In this case, we identified two compound heterozygous mutations in a case of late-onset severe Pompe's disease, who required prolonged ventilator support shortly after presentation, which improved with high protein diet and enzyme replacement therapy (ERT).

The first mutation at c.444C>G (p.Tyr148*) in exon 2 is a novel mutation and has not been previously reported in both HGMD and Pompe's Disease Mutation Database. The second mutation is at c.2238G>C (p.Trp746Cys) in exon 16. These 2 compound heterozygous mutations found in this patient are predicted to reduce the enzyme activity of acid *α*-glucosidase. Compound mutation of c.2238G>C (p.Trp746Cys) in exon 16 was previously reported in juvenile onset Pompe's disease patients as a sequence change of unknown pathogenic significance causing diminished enzyme activity [6]. Yang et al. [4] reported a group of 15 late-onset Chinese patients with symptom onset in their second decade of life with rapid disease progression. Mutation analysis in these patients revealed 2 dual mutations in the GAA gene c.(1935C>A; 1726G>A) (p.(D645E; G576S)) and c.(2238G>C; 1726G>A) (p.(W746C; G576S)) which represented 66.5% of the mutated chromosomes. Interestingly, Chinese patients with late-onset Pompe's disease displayed earlier symptom onset and faster disease progression as compared to the Dutch [7] and German [8] patients. They also have a poorer treatment response to recombinant human GAA. Yang attributed the aggravated phenotype observed in this group of patients to the lack of c.-32-13T>G (IVS1-13T>G) mutation and the low prevalence of the p.(W746C; G576S) and p.(D645E; G576S) mutations. The c.-32-13T>G mutation was found in 79% of the patients with Caucasian origin and reduces the fidelity of GAA mRNA splicing to 10% of the original amount [7, 9]. The residual activities correlated with the later age of onset and slower disease progression in late-onset Pompe's disease. However, the disease severity did not correlate with enzyme activity. The c.-32-13T>G mutation which is associated with a milder course of disease has a broad variability in the decline of locomotive and respiratory function [10]. Both Yang et al. [4] and L. Wan et al. [6] have reported the association of p.W746C mutation with earlier onset of symptoms in late-onset Pompe's disease, including one homozygous patient who showed onset of symptoms at the age of 10. In our case, the patient's symptom onset was at the age of 23 when she first experienced progressive proximal muscles weakness causing her to have difficulty in climbing stairs, squatting, and getting up. Phenotypically, she had weak axial and proximal limb muscles that was worse in the lower limbs with poor chest expansion and subsequently developing decompensated respiratory failure necessitating ventilatory support. This patient's novel mutation showed good initial response to high protein diet and later on enzyme replacement therapy though having severe disease but when interrupted it showed deterioration in motoric functioning and respiratory need.

Current management of adult-onset Pompe's disease is to reduce the glycogen deposition in skeletal muscle via dietary modification or enzyme replacement therapy. Data is available to support the use of high protein diet in adult-onset Pompe's disease [11, 12]. Underlying energy deficiency in Pompe's disease produces a chronic catabolic state with the potential of significantly impacting the skeletal muscles function and preservation [13]. This low carbohydrate and high protein intake is aimed at reducing muscle catabolism, preventing striated muscle wasting which compromises physical performance and leads to respiratory failure. Before the introduction of enzyme replacement therapy, the results of high protein therapy in adult-onset Pompe's disease had been encouraging. This diet, in combination with programmed exercise, reverses muscle glycogen accumulation and has been shown to retard the rate of clinical deterioration. This is also reported to be beneficial in patients with respiratory insufficiency [12]. However, only 25% of all reported subjects showed an improvement of muscle or respiratory function after a high protein diet [14]. This is mainly due to poor compliance with the large amount of protein diet required and the consequence of weight gain.

To date, enzyme replacement therapy (ERT) with alglucosidase alfa is the only treatment available for late-onset Pompe's disease in adults. Clinical outcome data implied that ERT stabilized neuromuscular deficits over 1 year [15] and led to an increase in the predicted forced vital capacity [16]. The late-onset treatment study (LOTS) shows that the benefits of ERT in treatment group (alternate week, 20 mg/kg dose) seen during the first 26 weeks of therapy and maintained during the entire 78 weeks of the study [16]. LOTS trial shows a modest but statistically significant benefit in the 6-minute walking test. However, this benefit does not translate into functionally significant improvement. This has raised the possibility that alglucosidase dose of alternate week, 20 mg/kg might be insufficiency in adult late-onset Pompe's patient. The currently prescribed alglucosidase dose was based on the clinical benefits in infantile-onset Pompe's disease [17].

Systematic review by Toscano and Schoser of 368 late-onset Pompe's disease patients on clinical efficacy, safety, and tolerability of ERT with alglucosidase alfa (most of them 20 mg/kg every other week) shows that at least two-thirds of them stabilised or had improved creatine kinases and muscular and/or respiratory function [18]. However, not all responders continue to show improvement with longer duration of ERT. 16.7% of those patients treated for >36 months showed decline in mean 6-minute walking test from the baseline. Meanwhile, although most patients showed improvement in FVC after >12 months of ERT, treatment for >24 months was not associated with any further improvement. The recently published prospective international observational study on the impact of enzyme replacement therapy on survival in adults with Pompe's disease which included 283 adult patients demonstrated the significant positive effects of ERT on survival, supporting its beneficial impact in adult patients [19]. This beneficial effect of ERT on survival is likely to be related to its positive effect on pulmonary function because most adult patients eventually die of respiratory failure. These findings reinforce what we see clinically in our patient with Pompe's disease who continues to survive without invasive ventilation even after 8 years as long as the treatment is not interrupted and also highlight that patients with this current mutation despite being very preliminary suggest a beneficial response to ERT.

In conclusion, we report a Malaysian Chinese lady with late-onset glycogen storage disease type II due to two compound heterozygous mutations. Genetic analysis revealed a novel mutation at c.444C>G (p.Tyr148*) in exon 2. This patient has typical clinical manifestation of late-onset Pompe's disease with proximal muscular weakness and rapid progression to respiratory insufficiency without cardiac complication. Early identification of the disease and treatment with high protein diet and enzyme replacement therapy in patients with this novel mutation improve muscle function and have beneficial impact on survival.

## Abbreviations

ERT: Enzyme replacement therapy
GAA: Acid *α*-1,4-glucosidase
HGMD: Human Gene Mutation Database
LOTS: Late-onset treatment study
MRC: Medical Research Council
MUAPs: Motor unit action potentials
rhGAA: Recombinant human acid *α*-glucosidase.
